# What frail, older patients talk about when they talk about self-care—a qualitative study in heart failure care

**DOI:** 10.1186/s12877-023-04538-1

**Published:** 2023-12-07

**Authors:** Jeanette Eckerblad, Leonie Klompstra, Linda Heinola, Sandra Rojlén, Nana Waldréus

**Affiliations:** 1 Department of Neurobiology, Care Sciences and Society, Division of Nursing, Karolinska Institutet, Stockholm, Sweden; 2 Department of Health, Medicine and Caring Sciences, Linköping University, Linköping, Sweden; 3 Theme Inflammation and Aging, Nursing Unit Aging, Karolinska University Hospital, Stockholm, Sweden; 4 Theme Women’s Health and Allied Health Professionals, Medical Unit Occupational Therapy & Physiotherapy, Karolinska University Hospital, Stockholm, Sweden

**Keywords:** Frailty, Self-care, Sleep, Multimorbidity, Heart failure

## Abstract

**Background:**

Self-care plays a crucial role in the management of heart failure (HF) and is especially important for older patients who are frail. However, there is limited knowledge about how frail, older patients with HF perceive and experience self-care. Therefore, the aim of this study was to describe the experiences of self-care among frail, older patients with HF.

**Methods:**

A qualitative descriptive design with semi-structured interviews with frail, older patients diagnosed with HF (*n* = 19; median age 82 years). Thematic analysis, guided by Braun and Clarke, was used to analyse the data.

**Results:**

Two main themes emerged from the analysis: 1) “To maintain my health,” encompassing various aspects such as hygiene practices, engaging in physical activity, medication adherence, following a healthy diet, and ensuring adequate rest; and 2) “To maintain my well-being and happiness,” highlighting the importance of hobbies, maintaining independence, participating in social activities, and creating a supportive environment.

**Conclusion:**

This study provides valuable insights into the perspectives of frail, older patients with HF regarding self-care. It was observed that older patients often associate self-care with general well-being, hygiene, and happiness. Clear communication between healthcare providers and patients is essential to align different perspectives on self-care and ensure that self-care plans are tailored to individual needs. Moreover, addressing the emotional well-being and happiness of patients should be prioritized, as these factors play a significant role in promoting self-care adherence.

**Supplementary Information:**

The online version contains supplementary material available at 10.1186/s12877-023-04538-1.

## Introduction

Self-care plays a crucial role in the managementof heart failure (HF) and holds a central position in patient care. Nurse-led self-care interventions have shown improvements in self-care management, self-care maintenance, and self-efficacy and a reduction in depression [1]. Patients with HF who engage in effective self-care experience lower mortality and readmission rates, improved psychological well-being, and a higher quality of life [2–8]. Orem defined self-care as “the practice of activities that individuals initiate and perform on their own behalf in maintaining life, health, and well-being” [9] (p117). For patients with chronic conditions, it is defined as follows: “self-care is a process of maintaining health through health promoting activities and managing illness” [10] (p195). Specific self-care practices for HF include, for example, engaging in regular physical activity, adhering to medication regimens, monitoring symptoms, taking additional diuretics as prescribed, and seeking medical advice from a healthcare provider [11].

In patients with HF, adherence to crucial HF self-care behaviours is typically low, and decreased self-care ability is associated with older age and frailty [12–14]. Frailty is a condition characterized by the decline of organ systems, adverse clinical outcomes, changes in health status and experiences of losses in social and socioeconomic aspects of life [12, 15, 16]. Frail, older patients with HF are particularly susceptible to frequent hospitalization and higher mortality rates [12, 13, 17, 18]. They may also experience difficulty in independently carrying out the essential self-care practices specific to HF [13]. Having HF increases the risk of developing frailty by up to six times compared to other populations. It is estimated that 45% of patients with HF meet the clinical definition of frailty [19–21].

Previous studies have demonstrated that patients with HF who had higher scores on the social component of frailty exhibited better self-care abilities [22–25]. Moreover, research has indicated a correlation between higher frailty scores and lower self-care capabilities [13, 26]. Additionally, physical and psychological frailty have been shown to have a negative impact on self-care capabilities in older patients with HF [23–25].

Frail patients with HF have been traditionally underrepresented in clinical trials and research studies due to their vulnerability and exclusion criteria. This limits our understanding of their unique self-care needs, preferences, and challenges. By assessing self-care understanding and experiences in frail, older patients with HF, we can gather valuable information and insights that can provide healthcare providers with a better understanding of how to support self-care in frail, older patients with HF. Therefore, the aim of this study was to describe the experiences of self-care among frail, older patients with HF.

## Methods

### Design and research team

This study had a qualitative descriptive design relying on individual and semi-structured interviews. The consolidated criteria for reporting qualitative research (COREQ, supplementary material) was used for transparency [27]. The research team consisted of assistant professors JE, NW, and LK, an associate professor, all with previous experience within the research field of older people with chronic diseases and experience in designing, analysing, and writing qualitative studies. Two specialist nursing students (LH and SR) participated in the study as part of their training.

### Setting and participants

Data were collected at the Department of Geriatrics at a middle-sized hospital in Stockholm County, Sweden, in 2021 during a six-month study period (May to November). Eligible patients had been diagnosed with HF, had the ability to communicate in Swedish, were 65 years of age or older and were frail. Purposeful sampling [28] was used to achieve a sample comprised of HF patients of different ages and frailty levels, as measured by the Clinical Frailty Scale [29]. This scale includes frailty levels from 1 (very fit) to 9 (terminally ill) [29]. In this study, we included older patients with HF with a frailty level between 4 and 6. A frailty level of 4 is described as a person who is vulnerable but not dependent on help from others and who often experiences symptoms that limit their activities; level 5 is described as a person who is mildly frail, who needs help or support with, e.g., heavy housework, transportation; and level 6 is described as a person who is moderately frail who needs help with, e.g., all outdoor activities, all housework, and with bathing/hygiene.

### Recruitment

Patients were screened by one of the authors (SR) by monitoring the enrollment of patients in the department through electronic medical records. A total of 130 patients were diagnosed with HF and were 65 years of age or older during the study period. To identify patients with frailty levels 4 to 6, the author (SR) measured frailty with assistance from clinicians by using the Clinical Frailty Scale [29]. Eligible patients were approached face-to-face for study participation by the author (SR) a few days before their discharge from the hospital. They were invited to participate and received oral and written information about the study.

### Data collection

Data on patient characteristics were collected at the hospital by one of the authors (SR). Sociodemographic data were collected with a questionnaire. Well-being was measured using Cantril´s ladder, where patients were asked to rate their sense of well-being on a ladder, ranging from (0) worst possible life to (10) the best possible life [30]. Clinical data, such as ejection fractions and the New York Heart Association’s functional classification, were retrieved from patients´ medical records. Comorbidities were also collected from the medical records by using the Charlson comorbidity index, scoring from 0 (no comorbidities) to a maximum of 37 [31].

Interviews were conducted by one of the authors (SR), with support and guidance from two senior researchers (NW & JE). The interviewer was not involved in the patient’s clinical care. All interviews took place in a secluded room at the hospital with only the patient and the interviewer present. During data collection, the interviewer used a mask and social distancing due to the COVID-19 pandemic. The older, frail patients with HF were informed that they could take a break during the interview if they needed to. They were also offered something to drink if they got thirsty.

A semi-structured interview guide was used for data collection (supplementary material). The first interview was pilot tested, and no adjustments were made. The pilot interview was included in the data analysis. They were asked for example, what they think about self-care, have they received advice or/and information about self-care, do they feel they do self-care in their daily life, and what they routinely do to take care of themselves (supplementary material). Participants were asked to talk freely, and for clarity, follow-up questions were asked, e.g., “please, tell me more; describe more about that; why; how”. We concluded recruitment when sufficient information power was achieved after 19 interviews. Information power relates to the specificity of the aim, purposive sampling, quality and depth of the interviews, and strategies of the analysis [32]. All interviews were audio-recorded and transcribed verbatim by a professional transcriber. Confidentiality was ensured by giving a code number to each interview transcript. The interviews were analysed once all interviews had been completed.

### Data analysis

Two of the authors analysed data (JE and LK) using inductive thematic analysis to identify and find patterns in the data [33]. In the first step the authors analysed the transcripts separately (i-iii) and in the second step the authors discussed and pooled the analyses together (iv-vi). The process of the analysis was as follows: (i) first, each author read the transcribed interviews several times to become familiarised with the data, making notes about initial ideas; (ii) interesting content relevant to the study’s aim was coded systematically in the data; and (iii) codes were organised into potential and relevant themes. All authors participated in a discussion to compare findings and recurring patterns. An initial thematic map was constructed: (iv) the two authors responsible for the analysis went back to the original data to review, name, and define the themes to ensure that the themes were clear in relation to the codes; (v) details were refined to generate clear names for each theme and each code. The final structure was created after all authors had checked and reviewed the data: (vi) producing results with the selected descriptions.

## Results

Fifty-five patients were eligible to participate in the study and a total of 19 patients agreed to participate. The median (25^th^ and 75^th^ percentiles) age of the 19 frail patients with HF was 82 (75 and 85) years, and all were born in Scandinavia. Of these, 13 patients (68%) were men. Seven patients with HF had a frailty level of 4, eight had a frailty level of 5, and four had a frailty level of 6. Eleven patients (58%) did not have home care or home healthcare services. The median (25^th^—75^th^ percentiles) time since HF diagnosis was 4 years (3–7). Ten patients (53%) were married or were living with someone, and 17 (89%) had children (Table 1).

**Table 1:** Characteristics of 19 frail, older patients with heart failure

**Age** (years), median (25^th^ and 75^th^ percentiles) and range	82 (75–85) 67 to 90
**Female gender**, n (%)	6 (32%)
**Married or living together**, n (%)	10 (53%)
**Home health care**, n (%)	8 (42%)
**Education**, n (%)	
Primary school	9 (47%)
Secondary school	4 (21%)
Higher education	6 (32%)
**Time since diagnose** (years), median (25^th^- 75^th^ percentiles)	4 (3—7)
**Ejection fraction %**, mean ± SD	43 ± 11
**NYHA class**, n (%)	
II	4 (21%)
III	11 (58%)
IV	4 (21%)
**Frailty level**, n (%)	
4: vulnerable	7 (37%)
5: mildly frail	8 (42%)
6: moderately frail	4 (21%)
**Comorbidities, n (%)**	
Diabetes	14 (74%)
Renal disease	14 (74%)
Malignant tumour	10 (53%)
Myocardial infarction	7 (37%)
Chronic pulmonary disease	6 (32%)
Cerebrovascular disease	5 (26%)
Mild liver disease	3 (16%)
Moderate to serious liver disease	2 (11%)
Metastatic, solid tumour	2 (11%)
Rheumatic disease	1 (5%)
Leukaemia	1 (5%)
**Well-being**^a^**,** mean ± SD	6 ± 2

*NYHA class* New York Heart Association Classification

^a^Well-being measured with the Ladder of Life were patients asked to rate their sense of well-being on a ladder, ranging from: (0) worst possible life to (10) the best possible life [30]

The most common comorbidities were diabetes (74%; *n* = 14), renal disease (74%; *n* = 14), and cancer (53%, *n* = 10). Approximately one-third of the patients also previously had a myocardial infarction (37%; *n* = 7), chronic pulmonary disease (32%; *n* = 6) and cerebrovascular disease (26%; *n* = 5). The Charlson mean score was 8 ± 4 (minimum score 3, and maximum 17). The mean level of well-being was 6 ± 2 (Table 1).

**Fig. 1:**
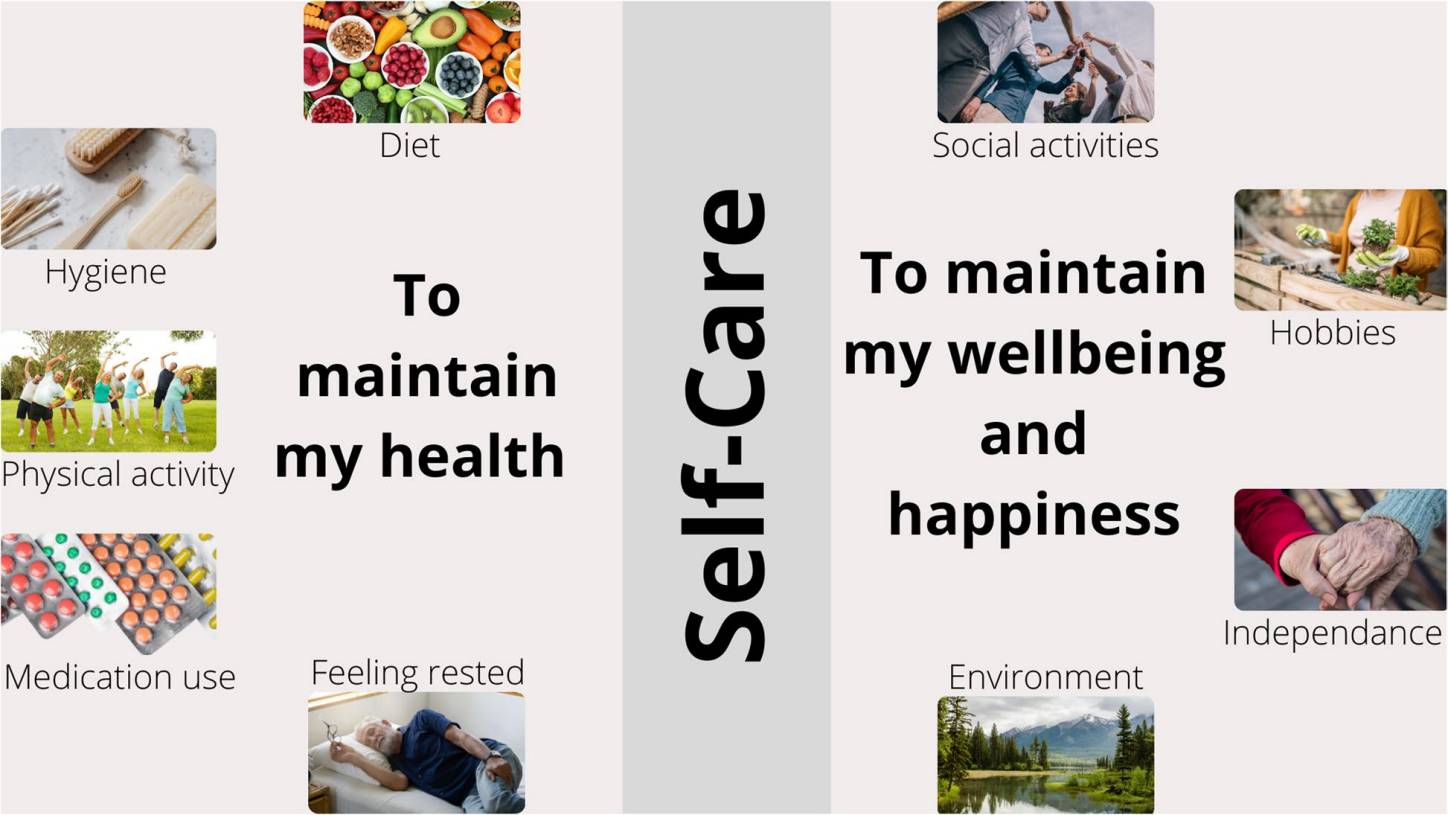
Frail, older patients with heart failure described self-care as a means of maintaining their health, well-being, and happiness

The mean time for the interviews was 27 min ± 12. Self-care in frail older people with HF was described by two main themes: (1) *to maintain my health* and (2) *to maintain my well-being and happiness *(Fig. 1)*.* The theme “to maintain my health” emerged from five codes: *hygiene, physical activity, medication use, diet* and *feeling rested.* The second theme, “to maintain my well-being and happiness”, emerged from four codes: *hobbies, independence, social activities,* and* environment.*

### To maintain my health

Engaging in good hygiene practices, such as taking regular showers, brushing teeth, and taking care of one's feet, was identified as an essential aspect of self-care for maintaining health. Additionally, participating in physical activities such as daily walking, regular cycling, or engaging in sports was emphasized as an important self-care activity.

**Man, 81 years of age, vulnerably:** *I'm trying to be as physically active as I possibly can. Me and my wife do Nordic walking almost every day.*

**Woman, 85 years of age, moderately frail:** *I'll try to stay physically active, I'm not exercising, but I try to stay physically active and eat reasonably healthy. That's what I try to do, by preparing my own food.*

However, these self-care practices were often mentioned in the past tense, indicating that while participants acknowledged their importance, they were no longer able to perform them to the extent they desired. This limitation was attributed to symptoms such as dizziness, shortness of breath, or pain. Additionally, physical and visual impairments pose additional challenges in maintaining their health.

**Woman, 79 years of age, mildly frail:** *I used to walk quite a lot. But then I've had heart surgery with by...what's it called? Bypass surgery. And after that I have become weaker and experience more shortness of breath.*

When encountering symptoms specific to their condition, whether heart-related or otherwise, patients sought assistance from their healthcare providers for support. Patients with HF made efforts to establish a daily routine to ensure that they knew which medications to take and when. However, adjusting the doses of various medications on their own at home was perceived as challenging.

**Woman, 73 years of age, vulnerable:*** For over a year I took the medications and injections and did all of that by myself. And that worked quite well. Yes, it worked very well. But, in the end, I stood there one day and thought…did I take that medicine? Did I take all the tablets? So, I thought, no, this is not for me. So, because then, if you do not know if you have done it right or wrong, then you should not go down this path.*

While maintaining a healthy diet was considered an essential self-care behaviour, patients also emphasized the significance of consuming foods and beverages they enjoyed and that were prepared at home. Furthermore, patients expressed the importance of having a good night's sleep and taking rest or relaxation when necessary for maintaining good health. They believed that feeling well rested could facilitate the process of taking care of themselves and attending to the needs of others throughout the day.

### To maintain my well-being and happiness

Patients with HF viewed self-care as engaging in activities that promoted their overall well-being and happiness. However, these activities did not necessarily pertain to the management of their HF condition. Pursuing hobbies such as reading books, knitting, or doing crosswords were mentioned as examples of activities that brought them joy and contributed to their happiness.

**Man, 90 years of age, vulnerably***: I'll read a little bit every now and then. And I read the news aloud to my wife, we both find the news interesting.*

Patients with HF expressed the importance of maintaining independence in their self-care activities. They found happiness in being able to perform these activities on their own. However, they also acknowledged that certain activities became challenging as their abilities declined, and they started relying on others for assistance.

**Woman, 79 years of age, mildly frail:*** I think I perform self-care. And I am very happy, as long as I can do this myself. Because unfortunately the day will come when I won't be able to do this by myself anymore.*

Patients with HF expressed a sense of privilege when they received assistance from others, be it their family or significant others. However, they emphasized the importance of the help being offered voluntarily rather than having to ask for it, as it made them feel burdensome. Additionally, some patients also had caregiving responsibilities towards other family members or significant others, which consumed their time and hindered their ability to engage in the self-care activities they desired.

Socializing and participating in social activities, such as meeting with family and friends, were deemed important for patients. They recognized the value of social interactions and highlighted the positive impact it had on their well-being and happiness.

**Woman, 67 years of age, mildly frail:** *I like to meet my old friends, we have known each other for many years. We go out for lunch sometimes or go see a movie together.*

However, currently, the frequency of social activities has decreased for these patients due to their advanced age. They expressed a sense of being distanced from the overall societal togetherness, attributing it to the generation gap and the perception that their age separates them from the rest of society.

**Man, 85 years of age, mildly frail:*** I don't know if I'm interpreting the situation correctly or not, but I never see anyone from the younger generation trying to connect with us older people, … everyone seems to be in a hurry all the time.*

The restrictions on social contact imposed during the COVID-19 pandemic exacerbated feelings of loneliness among patients with HF. The inability to participate in social activities further contributed to their low mood and hindered their ability to engage in the self-care practices they were aware of and should have undertaken.

**Woman, 70 years of age, moderately frail:** *I have been a little too inactive during the corona period. I think for sure that when it gets a little better with the corona, I will probably become more active. Of course, many times you think when you are inactive all day, that you would like to live in a nursing home. So that you...could go out and sit in a common living room and see other people and be able to talk to them.*

Being able to change their environment, whether by going on holiday, spending time in nature, or visiting their summer house, was also recognized as an important aspect of self-care that brought about a sense of well-being and happiness for patients with HF.

## Discussion

Frail, older patients with HF described self-care as a means of maintaining their health, well-being, and happiness, aligning with the definition proposed by Riegel et al. [10] According to their perspective, self-care involves engaging in health-promoting activities and managing their illness. In this study, participants mentioned self-care behaviours such as physical activity, maintaining a healthy diet, and adhering to prescribed medications for self-care maintenance. However, monitoring symptoms (e.g., weight monitoring) and symptom management (e.g., contacting healthcare providers when symptoms arise) were not emphasized when discussing self-care.

Interestingly, this study revealed self-care behaviours that are not commonly expressed in other research, including sleep hygiene activities and maintaining good personal hygiene practices (e.g., regular showers, brushing teeth). Existing research indicates that patients with HF often experience unsatisfactory and insufficient sleep [34], particularly older adults, who have a higher prevalence of insomnia (44%) than younger patients (31%) [35]. A literature review suggests a possible association between sleep quality, self-care, and medication adherence [36]. The practical management recommendations of the Heart Failure Association of the European Society of Cardiology recognize the significance of sleep quality in patients with HF, recommending assessment and intervention for sleeping problems, including addressing underlying causes such as fluid overload, anxiety, and depression. When patients experience insomnia, they could begin with sleep hygiene [11]. Sleep hygiene, which involves adopting behavioural and environmental recommendations for healthy sleep, can serve as an initial approach when patients experience insomnia [11, 37].

Hand hygiene is crucial in combating infectious diseases among older adults, but inadequate hand hygiene practices have been associated with lower health literacy. Good oral care hygiene in older adults can reduce oral infections and related complications in older adults [38]. Foot care is seen as especially important in patients with diabetes to prevent ulcers or infections in the foot. Given that 74% of the participants in our study also had diabetes, foot care emerged as an important aspect of self-care, particularly in preventing ulcers and infections.

Self-care was also expressed as activities that contribute to well-being and happiness. Engaging in hobbies and social interactions and having the freedom to change environments were cited as factors that promote happiness. Independence in daily activities, such as preparing meals, was deemed vital for self-care. Although Riegel’s definition [10] of self-care in patients with HF did not explicitly mention well-being, patients in this study emphasized the pursuit of happiness as an integral part of self-care. Other research on aging supports the notion that a variety of social and leisure activities among older adults predict better health and well-being, buffering against the impact of spousal loss, functional impairment, and poor family support [39].

Maintaining independence in performing activities of daily living was perceived as a contributor to well-being and happiness among frail, older patients with HF. Other studies indicate that increasing difficulties in daily activities for HF patients correlate with higher mortality and hospitalization risks [40]. Assessing patients' ability to perform activities of daily living is thus important in HF care.

Social activities were also recognized as an important component of self-care in our study. Patients reported feeling lonely due to a generation gap with society, and the COVID-19 pandemic further limited their social interactions. Research highlights the significance of assessing loneliness in cardiac disease patients, as it strongly correlates with poor patient-reported outcomes and mortality. Additionally, living alone, experiencing loneliness, and lacking social support have been associated with hospital readmissions in frail HF patients [17, 41]. Support from family is crucial for individuals enduring chronic illness [42]. In our study, maintaining contact with family and friends through phone calls and spending time with them were described as essential aspects of maintaining well-being, happiness, and self-care. However, patients expressed a desire not to burden their family members while appreciating the assistance offered. Previous research suggests that healthcare should support patients' social interactions as part of their self-care, recognizing the significance of empathy, respect, and engagement between patients, caregivers, and family members [42, 43]. Focusing on these aspects is valuable in HF care [44].

Participants in our study reported that changing their environment, such as going on holidays or visiting a summerhouse, played a significant role in their well-being and happiness. It is important to note that HF admission rates are typically lower during holidays [45]. However, research shows a higher admission rate immediately after holidays, possibly due to emotional stressors, reduced exercise, and delayed medical care during holiday periods. Patients should be made aware of the potential risks associated with decreased self-care during holidays [45]. Participants in our study also considered being physically active as an important aspect of self-care. However, previous studies have shown that patients with HF may face physical barriers to engaging in adequate exercise [46]. For older and more frail patients with HF, individualized support may be necessary to facilitate physical activity.

## Strengths and limitations

To the best of our knowledge, this study is one of the first to explore the concept of self-care as described by frail, older patients with HF. Through interviews, we gained insights into the experiences and perspectives of patients regarding self-care. To ensure the reliability of the interview data, all authors reviewed the analysis process and discussed the findings within the research group. The participants in this study varied in age, ranging from 67 to 90 years. However, it is important to acknowledge the limitations of this study. Our goal was to recruit participants across all three levels of frailty (vulnerable, mildly, and moderately frail), but only four participants fell into the moderately frail category. Moreover, the participants in our study had multiple chronic diseases in addition to HF. The presence of multimorbidity can lead to various symptoms that may have influenced the self-care practices observed [47].

## Conclusions

In the context of HF care, it is important to recognize that older patients often have a broader understanding of the concept of self-care, encompassing general aspects such as hygiene, well-being, and happiness. On the other hand, healthcare professionals in clinical practice tend to associate self-care primarily with disease-related aspects, such as responding to symptoms when they arise. To ensure effective support for self-care, it is crucial to clearly communicate how self-care is meant to be understood in the clinical context to both patients and caregivers. Additionally, addressing well-being and happiness is essential, as these factors contribute to adherence to self-care practices. Therefore, in clinical practice, it is important to assess and communicate about activities of daily living and independence, as these aspects appear to play a significant role in self-care among frail, older patients with HF.

### Supplementary Information

**Additional file 1.** Consolidated criteria for Reporting qualitative studies (COREQ): 32-item checklist.

**Additional file 2.** Interview guide about self-care for older, frail persons with heart failure.

## Data Availability

The datasets used and/or analysed during the current study are available from the corresponding author on reasonable request.

## Abbreviations

COVID-19: Coronavirus disease of 2019
HF: Heart failure

## References

[1] Huang Z, Liu T, Chair SY (2022). Effectiveness of nurse-led self-care interventions on self-care behaviors, self-efficacy, depression and illness perceptions in people with heart failure: a systematic review and meta-analysis. Int J Nurs Stud.

[2] Lee CS, Bidwell JT, Paturzo M (2018). Patterns of self-care and clinical events in a cohort of adults with heart failure: 1 year follow-up. Heart Lung.

[3] Riegel B, Dickson VV, Faulkner KM (2016). The situation-specific theory of heart failure self-care: revised and updated. J Cardiovasc Nurs.

[4] Riegel B, Moser DK, Anker SD (2009). State of the science: promoting self-care in persons with heart failure: a scientific statement from the American Heart Association. Circulation.

[5] Heo S, Moser DK, Lennie TA (2008). Gender differences in and factors related to self-care behaviors: a cross-sectional, correlational study of patients with heart failure. Int J Nurs Stud.

[6] Joekes K, Van Elderen T, Schreurs K (2007). Self-efficacy and overprotection are related to quality of life, psychological well-being and self-management in cardiac patients. J Health Psychol.

[7] Rohrbaugh MJ, Shoham V, Coyne JC (2004). Beyond the “self” in self-efficacy: Spouse confidence predicts patient survival following heart failure. J Fam Psychol.

[8] Lee CS, Westland H, Faulkner KM (2022). The effectiveness of self-care interventions in chronic illness: a meta-analysis of randomized controlled trials. Int J Nurs Stud.

[9] Orem DE (1991). Nursing: Concepts of practice.

[10] Riegel B, Jaarsma T, Strömberg A (2012). A middle-range theory of self-care of chronic illness. ANS Adv Nurs Sci.

[11] Jaarsma T, Hill L, Bayes-Genis A (2021). Self-care of heart failure patients: practical management recommendations from the Heart Failure Association of the European Society of Cardiology. Eur J Heart Fail.

[12] Uchmanowicz I, Wleklik M, Gobbens RJ (2015). Frailty syndrome and self-care ability in elderly patients with heart failure. Clin Interv Aging.

[13] Vidan MT, Martin Sanchez FJ, Sanchez E (2019). Most elderly patients hospitalized for heart failure lack the abilities needed to perform the tasks required for self-care: impact on outcomes. Eur J Heart Fail.

[14] Jaarsma T, Stromberg A, Ben Gal T (2013). Comparison of self-care behaviors of heart failure patients in 15 countries worldwide. Patient Educ Couns.

[15] Bell SP, Saraf AA (2016). Epidemiology of multimorbidity in older adults with cardiovascular disease. Clin Geriatr Med.

[16] Vetrano DL, Palmer K, Marengoni A (2019). Frailty and multimorbidity: a systematic review and meta-analysis. J Gerontol A Biol Sci Med Sci.

[17] Uchmanowicz I, Kuśnierz M, Wleklik M (2018). Frailty syndrome and rehospitalizations in elderly heart failure patients. Aging Clin Exp Res.

[18] Yang X, Lupón J, Vidán MT (2018). Impact of frailty on mortality and hospitalization in chronic heart failure: a systematic review and meta-analysis. J Am Heart Assoc.

[19] Khan H, Kalogeropoulos AP, Georgiopoulou VV (2013). Frailty and risk for heart failure in older adults: the health, aging, and body composition study. Am Heart J.

[20] Woods NF, LaCroix AZ, Gray SL (2005). Frailty: emergence and consequences in women aged 65 and older in the Women's Health Initiative Observational Study. J Am Geriatr Soc.

[21] Denfeld QE, Winters-Stone K, Mudd JO (2017). The prevalence of frailty in heart failure: a systematic review and meta-analysis. Int J Cardiol.

[22] Uchmanowicz I, Gobbens RJ (2015). The relationship between frailty, anxiety and depression, and health-related quality of life in elderly patients with heart failure. Clin Interv Aging.

[23] Li J, Han J, Luo N (2022). Frailty Affects Self-Care Behavior in Congestive Heart Failure. Clin Nurs Res.

[24] Mlynarska A, Golba KS, Mlynarski R (2018). Capability for self-care of patients with heart failure. Clin Interv Aging.

[25] Nakhjiri LZ, Darvishpour A, Pourghane P (2021). The relationship between frailty syndrome and self-care ability in the elderly with heart failure. J Educ Health Promot.

[26] Son YJ, Lee K, Kim BH (2019). Gender differences in the association between frailty, cognitive impairment, and self-care behaviors among older adults with atrial fibrillation. Int J Environ Res Public Health.

[27] Tong A, Sainsbury P, Craig J (2007). Consolidated criteria for reporting qualitative research (COREQ): a 32-item checklist for interviews and focus groups. Int J Qual Health Care.

[28] Patton MQ (2002). Qualitative research and evaluation methods.

[29] Rockwood K, Song X, MacKnight C (2005). A global clinical measure of fitness and frailty in elderly people. CMAJ.

[30] Cantril H (1965). The patterns of human concerns.

[31] Charlson ME, Pompei P, Ales KL (1987). A new method of classifying prognostic comorbidity in longitudinal studies: development and validation. J Chronic Dis.

[32] Malterud K, Siersma VD, Guassora AD (2016). Sample size in qualitative interview studies: guided by information power. Qual Health Res.

[33] Braun V, Clarke V (2006). Using thematic analysis in psychology. Qual Res Psychol.

[34] Pearse SG, Cowie MR (2016). Sleep-disordered breathing in heart failure. Eur J Heart Fail.

[35] Gau FY, Chen XP, Wu HY (2011). Sleep-related predictors of quality of life in the elderly versus younger heart failure patients: a questionnaire survey. Int J Nurs Stud.

[36] Spedale V, Luciani M, Attanasio A (2021). Association between sleep quality and self-care in adults with heart failure: a systematic review. Eur J Cardiovasc Nurs.

[37] Hauri PJ, Hauri PJ (1992). Sleep hygiene, relaxation therapy, and cognitive interventions. Case studies in insomnia.

[38] Coll PP, Lindsay A, Meng J (2020). The prevention of infections in older adults: oral health. J Am Geriatr Soc.

[39] Adams KB, Leibbrandt S, Moon H (2011). A critical review of the literature on social and leisure activity and wellbeing in later life. Ageing Society.

[40] Dunlay SM, Manemann SM, Chamberlain AM (2015). Activities of daily living and outcomes in heart failure. Circ Heart Fail.

[41] Christensen AV, Juel K, Ekholm O (2020). Significantly increased risk of all-cause mortality among cardiac patients feeling lonely. Heart.

[42] Nordfonn OK, Morken IM, Lunde Husebø AM (2020). A qualitative study of living with the burden from heart failure treatment: exploring the patient capacity for self-care. Nurs Open.

[43] Nordfonn OK, Morken IM, Bru LE (2019). Patients' experience with heart failure treatment and self-care-A qualitative study exploring the burden of treatment. J Clin Nurs.

[44] Heckman GA, McKelvie RS, Rockwood K (2018). Individualizing the care of older heart failure patients. Curr Opin Cardiol.

[45] Shah M, Bhalla V, Patnaik S (2016). Heart failure and the holidays. Clin Res Cardiol.

[46] Del Buono MG, Arena R, Borlaug BA (2019). Exercise intolerance in patients with heart failure: JACC State-of-the-Art Review. J Am Coll Cardiol.

[47] Meyerson KL, Kline KS (2009). Qualitative analysis of a mutual goal-setting intervention in participants with heart failure. Heart Lung.

