# Supplementary material for: What frail, older patients talk about when they talk about self-care—a qualitative study in heart failure care
Source: BMC Geriatr. 2023 Dec 7;23:818. doi: 10.1186/s12877-023-04538-1 (PMC10704742; doi:10.1186/s12877-023-04538-1)
Supplement: Supplementary file 2 — Additional file 2. Interview guide about self-care for older, frail persons with heart failure. [file 12877_2023_4538_MOESM2_ESM.docx]

Interview guide about self-care for older, frail persons with heart failure

| - Please tell me if you have received advice or/and information about self-care? - If so, did you feel that the advice was relevant for you and that helped you? - What advice did you get, please describe? - Can you tell me about what routines you use to take care of yourself in the best ways? - Can you tell, is there something you usually do to make you feel better? - When you think about self-care, what do you think about? - Is self-care something that you feel that you do in your daily life? - Can you tell, how confident do you feel about performing self-care? - What could make it easier for you to practice self-care? - Is there something that hinders you from practicing self-care? |
| --- |
